# Oral Kaposi Sarcoma With Clinical Features Suggestive of Concurrent Multicentric Castleman Disease in Advanced AIDS

**DOI:** 10.7759/cureus.116543

**Published:** 2026-09-19

**Authors:** Jamil Qiqieh, Imeelda Altone, Kareem Siada, Sonora Desai, Avery Mendelson

**Affiliations:** 1 Internal Medicine, Henry Ford Health System, Southfield, USA; 2 Internal Medicine, Michigan State University, Lansing, USA

**Keywords:** antiretroviral therapy, hiv/aids, human herpesvirus-8, immunosuppression, kaposi sarcoma, multicentric castleman disease

## Abstract

Kaposi sarcoma (KS) is an AIDS-defining malignancy associated with advanced HIV infection and human herpesvirus-8 (HHV-8). We report a case of biopsy-proven oral KS in a 31-year-old patient with advanced AIDS and clinical features suggestive of concurrent multicentric Castleman disease (MCD), highlighting the diagnostic complexity of overlapping AIDS-related conditions. We describe the clinical presentation, diagnostic evaluation, imaging findings, histopathologic features, and management of a 31-year-old man with advanced AIDS and antiretroviral nonadherence presenting with constitutional, gastrointestinal, and neurologic symptoms. Laboratory evaluation demonstrated profound immunosuppression, elevated HIV viral load, cytopenias, hepatocellular injury, and markedly elevated inflammatory markers. Physical examination revealed violaceous oral and cutaneous lesions. Infectious evaluation was largely unrevealing, while imaging demonstrated diffuse lymphadenopathy and inflammatory colitis. Histopathologic examination of an oral lesion confirmed KS with HHV-8 positivity. The combination of diffuse lymphadenopathy, systemic inflammation, and cytopenias raised concern for concurrent MCD. The patient was restarted on antiretroviral therapy (ART) and initiated on trimethoprim-sulfamethoxazole for *Pneumocystis jirovecii* pneumonia prophylaxis, with outpatient oncologic follow-up arranged. This case highlights the diagnostic complexity of advanced HIV infection, in which infectious, inflammatory, and malignant processes may coexist. Tissue diagnosis remains essential for distinguishing overlapping HHV-8-associated diseases and guiding management.

## Introduction

Kaposi sarcoma (KS) is an angioproliferative tumor caused by human herpesvirus 8 (HHV-8), also known as KS-associated herpesvirus (KSHV) [1]. Before the widespread availability of antiretroviral therapy (ART), up to 30% of people with AIDS developed KS [1,2]. Following the introduction of ART in the mid-1990s, the incidence of KS declined substantially in the United States [1,3]. Despite this decline, KS risk remains markedly elevated among people with HIV, and KS remains one of the most common malignancies in this population [1,3].

HHV-8 can be identified in tissue using immunohistochemistry for latency-associated nuclear antigen-1 (LANA-1), a marker of latent KSHV infection expressed in the nuclei of lesional spindle cells [1,4]. Histologically, KS is characterized by spindle-cell proliferation, slit-like vascular spaces, inflammatory infiltrates, extravasated erythrocytes, and hemosiderin deposition [1].

KS may involve the oral mucosa, gastrointestinal tract, lymph nodes, lungs, and other organs, with presentations ranging from indolent cutaneous patches to severe visceral disease [1]. The AIDS Clinical Trials Group (ACTG) staging system categorizes patients according to tumor extent (T), immune status (I), and systemic illness (S) [2,5]. Disease limited to the skin and/or lymph nodes with minimal oral involvement is classified as good-risk tumor stage (T0), whereas tumor-associated edema or ulceration, extensive oral disease, or visceral involvement is classified as poor-risk tumor stage (T1) [2,5].

Definitive diagnosis requires biopsy with histopathology and HHV-8 LANA-1 immunohistochemistry to distinguish KS from mimics such as bacillary angiomatosis and other vascular lesions [2,4]. In a retrospective Botswana cohort, clinical suspicion of KS had a positive predictive value of only 58%, emphasizing the importance of tissue confirmation [6]. The National Comprehensive Cancer Network (NCCN) recommends repeat biopsy when the initial histopathologic evaluation is nondiagnostic but clinical suspicion remains high [2].

Multicentric Castleman disease (MCD) is an HHV-8-driven B-cell lymphoproliferative disorder that, in people with HIV, presents with fevers, diffuse lymphadenopathy, hepatosplenomegaly, cytopenias, and markedly elevated inflammatory markers, and shows regressed, vascularized germinal centers with LANA-1-positive plasmablasts on lymph node biopsy [7,8]. Because MCD and KS are driven by the same virus, the two frequently coexist, with concurrent KS identified in 63% of MCD-involved lymph nodes among HIV-positive individuals, making HHV-8 LANA-1 immunohistochemistry central to identifying both [1,9].

We present a 31-year-old man with advanced AIDS related to antiretroviral therapy (ART) nonadherence who was found to have biopsy-proven oral KS, diffuse lymphadenopathy, imaging findings suggestive of colitis, and markedly elevated inflammatory markers, raising concern for possible concurrent MCD. This case illustrates the diagnostic complexity of advanced HIV disease characterized by profound CD4+ T-lymphocyte depletion (4 cells/μL), uncontrolled viral replication (HIV-1 RNA, 92,400 copies/mL), and an AIDS-defining malignancy, in which infectious, inflammatory, and neoplastic processes may coexist and produce overlapping clinical manifestations.

## Case presentation

A 31-year-old man with HIV/AIDS and reported ART nonadherence while prescribed Biktarvy presented with a seven- to 10-day history of generalized headache with photophobia, fevers, chills, watery diarrhea, daily nonbloody emesis, an episode of syncope, and blurry vision. His medical history was notable for *Pneumocystis jirovecii* pneumonia in 2024 and resolved hepatitis A in 2019.

Five days earlier, the patient had been admitted to an outside hospital with similar symptoms. At that time, cerebrospinal fluid (CSF) analysis was unremarkable, with glucose of 52 mg/dL, protein of 43 mg/dL, and 2 nucleated cells/mm³. Gram stain, cultures, cryptococcal antigen, and herpes simplex virus (HSV) polymerase chain reaction (PCR) testing were negative. His CD4 count was 4 cells/μL, with a CD4 ratio of 0.01.

On arrival, the patient appeared ill, with tachycardia at 113 beats per minute, a blood pressure of 99/63 mmHg, and a temperature of 99.8°F. Physical examination was notable for bilateral conjunctival injection, dry mucous membranes, bilateral anterior cervical lymphadenopathy, mild epigastric tenderness, and diffuse violaceous lesions involving the skin and oral mucosa, including the hard palate and left alveolar ridge (Figure 1). No meningeal signs were present. The lesions had previously been clinically attributed to eosinophilic pustular folliculitis at the outside hospital; however, no skin biopsy was performed.

**Figure 1:**
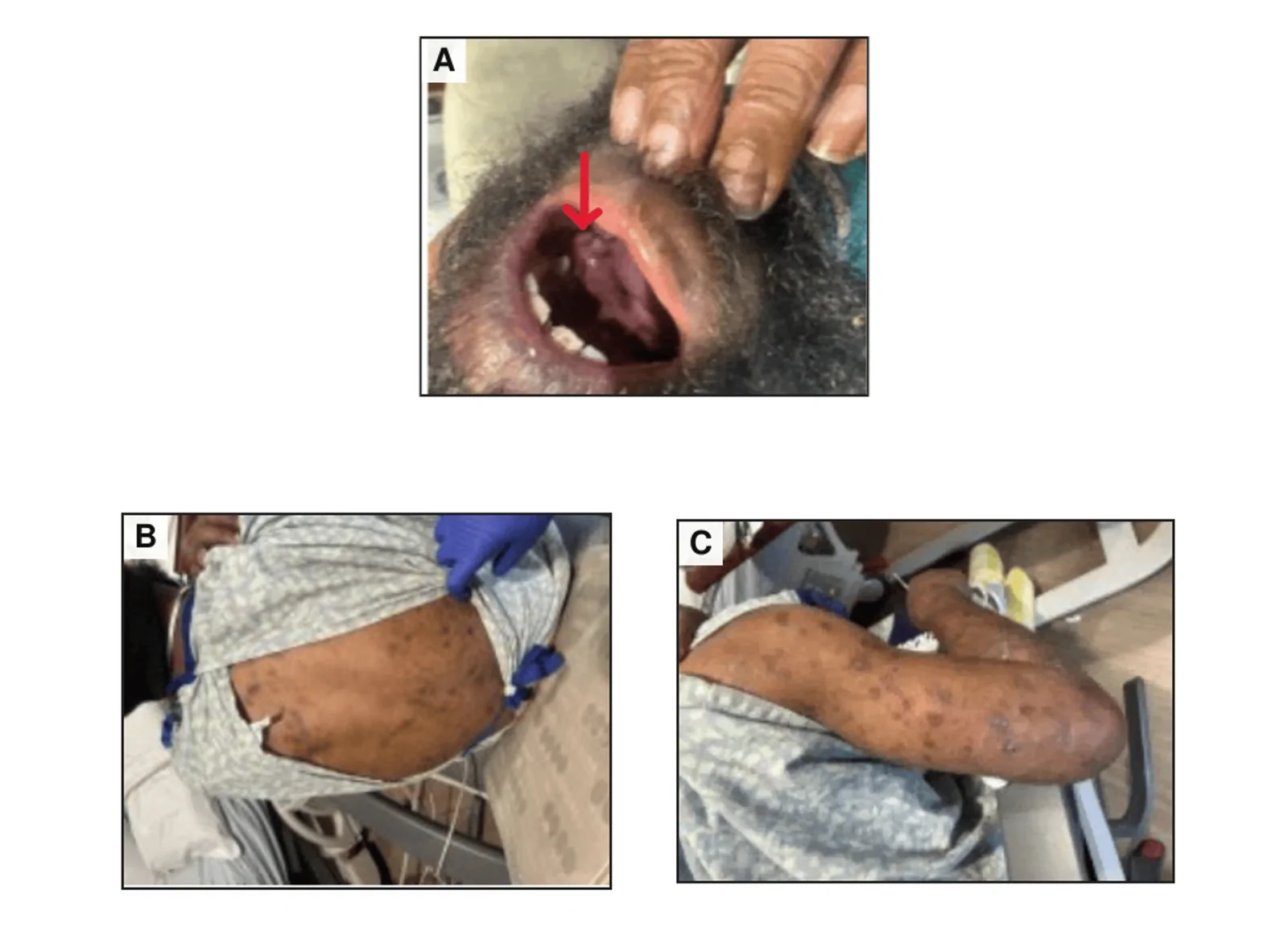
Clinical appearance of oral and cutaneous lesions Clinical photographs demonstrating a violaceous lesion involving the left alveolar ridge (A) and hyperpigmented cutaneous lesions involving the abdomen (B) and lower extremity (C). The left alveolar ridge lesion was subsequently biopsied and confirmed as Kaposi sarcoma. The cutaneous lesions were not biopsied.

Laboratory evaluation demonstrated profound lymphopenia, normocytic anemia, thrombocytopenia, acute kidney injury, acute hepatocellular injury, markedly elevated inflammatory markers, elevated lactate, and an elevated plasma HIV-1 RNA level (Table 1). The aminotransferase elevations represented a marked increase from normal values obtained five days earlier. Acetaminophen, ethanol, and salicylate levels were undetectable, and the acute hepatitis panel was negative.

**Table 1:** Laboratory findings

Laboratory test	Patient result	Laboratory reference range	Interpretation
White blood cell count	9.17 × 10³/µL	4.00–11.00 × 10³/µL	Normal
Hemoglobin	10.8 g/dL	13.5–17.5 g/dL	Low
Platelet count	120 × 10³/µL	150–400 × 10³/µL	Low
Absolute lymphocyte count, automated	0.09 × 10³/µL	1.00–5.00 × 10³/µL	Low
Absolute lymphocyte count, flow panel	0.07 × 10³/µL	1.00–5.00 × 10³/µL	Low
Lymphocytes	6%	19–48%	Low
Absolute CD4-positive T-helper count	4 cells/µL	>500	Markedly low
CD4-positive T-helper percentage	7%	37–63%	Low
HIV-1 RNA	92,400 copies/mL (4.97 log₁₀ copies/mL)	Not detected	Detected
Creatinine	1.9 mg/dL	0.6–1.2 mg/dL	High
Aspartate aminotransferase	939 U/L	10–40 U/L	High
Alanine aminotransferase	386 U/L	10–40 U/L	High
Alkaline phosphatase	203 U/L	20–120 U/L	High
Total bilirubin	2.2 mg/dL	0.1–1.2 mg/dL	High
Lactate dehydrogenase	946 U/L	135–225 U/L	High
Lactic acid	3.7 mmol/L	0.5–2.0 mmol/L	High

Computed tomography (CT) of the head was unremarkable. Repeat lumbar puncture revealed elevated protein of 200 mg/dL, glucose of 52 mg/dL, 79,000 red blood cells (RBCs)/mm³, and 57 white blood cells (WBCs)/mm³, including 94% polymorphonuclear cells (PMNs), 3% lymphocytes, and 3% monocytes. The pleocytosis was attributed to a traumatic tap, and the infectious diseases consultant recommended against empiric antibiotics. Extensive CSF infectious testing, including PCR for HSV, cytomegalovirus (CMV), enterovirus, varicella-zoster virus, and adenovirus, was negative. The repeat CSF findings are summarized in Table 2.

**Table 2:** Repeat cerebrospinal fluid findings

Laboratory test	Patient result	Laboratory reference range	Interpretation
White blood cell count	9.17 × 10³/µL	4.00–11.00 × 10³/µL	Normal
Hemoglobin	10.8 g/dL	13.5–17.5 g/dL	Low
Platelet count	120 × 10³/µL	150–400 × 10³/µL	Low
Absolute lymphocyte count, automated	0.09 × 10³/µL	1.00–5.00 × 10³/µL	Low
Absolute lymphocyte count, flow panel	0.07 × 10³/µL	1.00–5.00 × 10³/µL	Low
Lymphocytes	6%	19%–48%	Low
Absolute CD4-positive T-helper count	4 cells/µL	>500	Markedly low
CD4-positive T-helper percentage	7%	37%–63%	Low
HIV-1 RNA	92,400 copies/mL (4.97 log₁₀ copies/mL)	Not detected	Detected
Creatinine	1.9 mg/dL	0.6–1.2 mg/dL	High
Aspartate aminotransferase	939 U/L	10–40 U/L	High
Alanine aminotransferase	386 U/L	10–40 U/L	High
Alkaline phosphatase	203 U/L	20–120 U/L	High
Total bilirubin	2.2 mg/dL	0.1–1.2 mg/dL	High
Lactate dehydrogenase	946 U/L	135–225 U/L	High
Lactic acid	3.7 mmol/L	0.5–2.0 mmol/L	High

Contrast-enhanced CT of the abdomen and pelvis demonstrated colonic wall thickening from the descending colon to the rectum, bilateral inguinal and iliac chain lymphadenopathy with surrounding inflammatory stranding, nonspecific retroperitoneal lymphadenopathy, and incompletely characterized hepatic lesions (Figure 2). Abdominal ultrasound showed heterogeneous hepatic parenchyma consistent with nonspecific hepatocellular disease. Stool studies were positive for occult blood and lactoferrin. Stool culture was negative for *Salmonella*, *Shigella*, *Campylobacter*, and *Escherichia coli *O157:H7. Routine ova-and-parasite testing showed no ova, cysts, or parasites; however, the assay did not include specific testing for *Cryptosporidium*, *Cyclospora*, or *Microsporidia*, and these infections could not be definitively excluded. Syphilis screening was nonreactive; *Toxoplasma *and *Bartonella* serologies, urinary *Histoplasma *antigen, and QuantiFERON-TB Gold testing were negative. CMV IgG was positive, CMV IgM was negative, and plasma CMV DNA was detected below the lower limit of quantification (<35 IU/mL). Epstein-Barr virus (EBV) testing and acid-fast bacillus blood cultures to evaluate for disseminated *Mycobacterium avium* complex infection were ordered; however, no final results were available. Consequently, EBV-associated lymphoproliferative disease and disseminated *Mycobacterium avium* complex infection could not be definitively excluded.

**Figure 2:**
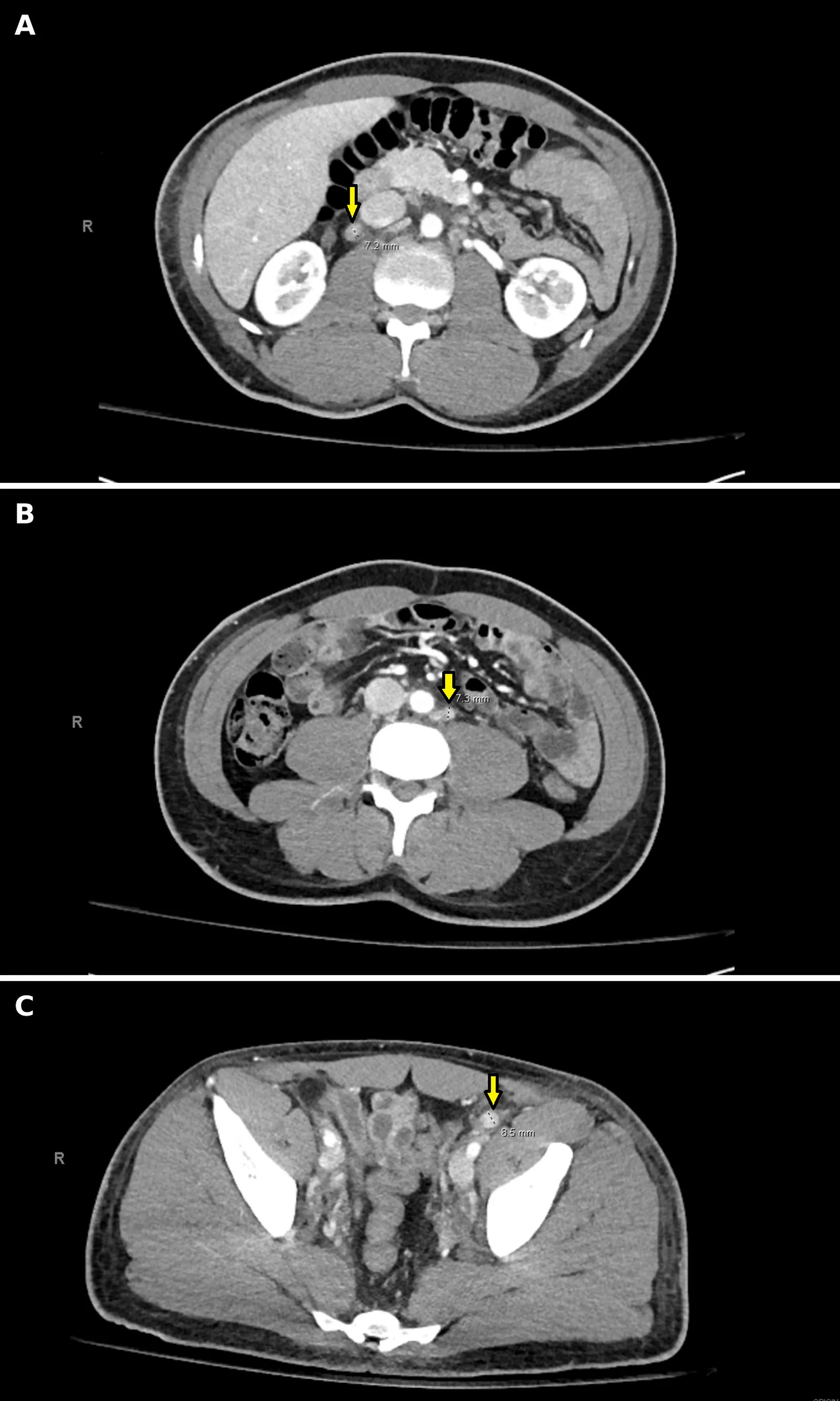
Axial abdominopelvic CT images Contrast-enhanced axial CT images demonstrating abdominopelvic lymphadenopathy at multiple levels. Yellow arrows indicate representative lymph nodes measuring 7.2 mm (A), 7.3 mm (B), and 8.5 mm (C).

The transaminitis improved during the hospital course (aspartate aminotransferase (AST) 939 → 113 U/L; alanine aminotransferase (ALT) 386 → 133 U/L) and was attributed to a likely medication-induced etiology. The acute kidney injury resolved with fluid resuscitation. During admission, Biktarvy was restarted, and prophylactic trimethoprim-sulfamethoxazole was initiated.

Given concern for KS, otolaryngology performed a biopsy of the left alveolar ridge lesion. Histopathology was compatible with KS, with immunohistochemical staining positive for HHV-8 and the endothelial markers CD31 and CD34.

The patient was discharged on Biktarvy and trimethoprim-sulfamethoxazole prophylaxis, with outpatient follow-up arranged with infectious diseases and internal medicine. Further outpatient evaluation of the diffuse lymphadenopathy and hepatic lesions with magnetic resonance imaging (MRI) was planned. Following pathologic confirmation of KS, the patient was also referred to hematology-oncology for chemotherapy evaluation. No lymph node biopsy was performed during the hospitalization; therefore, concurrent MCD could not be confirmed.

## Discussion

We present a man in his 30s with advanced AIDS and profound immunosuppression (CD4 count of 4 cells/μL and viral load of 92,400 copies/mL) who presented with biopsy-proven oral KS, acute hepatocellular injury, colonic findings suggestive of inflammatory colitis, and diffuse lymphadenopathy. His prior episode of *Pneumocystis jirovecii* pneumonia and subsequent presentation with a CD4 count of 4 cells/μL despite being prescribed Biktarvy highlights a critical failure in treatment adherence and the importance of addressing barriers to compliance in preventing AIDS-defining malignancies. This case illustrates how profound immunosuppression can produce a complex, multisystem presentation requiring simultaneous evaluation of infectious, malignant, and inflammatory etiologies.

The patient's recurrent headache with CD4 <50 cells/mm³ necessitated urgent evaluation for central nervous system (CNS) opportunistic infections, including cryptococcal meningitis, CNS toxoplasmosis, and tuberculous meningitis. Initial CSF analysis was relatively unremarkable (glucose 52 mg/dL, protein 43 mg/dL, 2 nucleated cells) with a negative infectious workup. A subsequent lumbar puncture demonstrated 57 WBCs (94% PMNs) with 79,000 RBCs, consistent with a traumatic tap. Standard correction methods, subtracting one WBC for every 500-1,500 RBCs, account for the apparent pleocytosis [10]. Nevertheless, these findings do not exclude meningitis. In advanced HIV, impaired cell-mediated immunity often produces attenuated inflammatory responses and deceptively normal CSF profiles, and over 50% of community-acquired meningitis cases in HIV-infected individuals have no identifiable etiology [10,11]. Meningitis therefore remains a key diagnostic consideration warranting close monitoring.

KS remains the most common AIDS-defining malignancy and is strongly associated with HHV-8 infection [12]. Although ART has dramatically reduced KS incidence, risk remains more than 30-fold higher in people with HIV [12]. A Center for AIDS Research Network of Integrated Clinical Systems (CNICS) cohort study (>20,000 people with HIV, 1996-2016) demonstrated that CD4 <200 cells/µL at ART initiation conferred a >6-fold increased KS risk, while HIV viral load >50,000 copies/mL conferred a >3-fold increase; conversely, every 10% increase in time with CD4 >350 cells/µL was associated with 24% lower KS risk [13]. This patient's advanced disease (T1 tumor stage, I1 immune status, S1 systemic illness per ACTG criteria) indicates a poor prognosis. Nasti et al. demonstrated that patients with T1S1 disease in the highly active ART (HAART) era had a three-year survival of 53%, compared to 88% in T0S0 disease (p = 0.0001) [14].

An important management consideration is the risk of immune reconstitution inflammatory syndrome (IRIS). KS-associated IRIS (KS-IRIS), characterized by unmasking or paradoxical worsening of KS following ART initiation, occurs in 6%-29% of patients with KS and HIV initiating ART in the US and Europe, with higher rates (18%-31%) in sub-Saharan Africa [15]. Risk factors include advanced tumor stage (T1), pretreatment viral load >5 log₁₀ copies/mL, detectable plasma HHV-8, and ART initiation without concurrent chemotherapy [15]. In this patient, profound immunosuppression, a viral load approaching 5 log₁₀ copies/mL, and ART reinitiation without concurrent chemotherapy heightened concern for KS-IRIS. Management includes systemic chemotherapy and supportive measures; ART should not be discontinued, and glucocorticoids are contraindicated due to their association with KS exacerbation (AIII) [3]. Close surveillance during immune reconstitution is essential given his extremely low baseline CD4 count.

Secondary hemophagocytic lymphohistiocytosis (HLH), which may complicate advanced HIV and associated opportunistic infections, was also included in the differential diagnosis [16]. In this patient, marked hyperferritinemia (6,167 ng/mL), anemia, thrombocytopenia, hepatocellular injury, and systemic inflammation raised this concern. However, a complete HLH evaluation was not performed; triglyceride and fibrinogen levels were not obtained, and a bone marrow biopsy was not performed. Consequently, the HLH-2004 criteria could not be fully assessed, and HLH remained unconfirmed [17].

The diffuse lymphadenopathy and bilateral lower extremity edema further raise concern for HHV-8-associated MCD, a lymphoproliferative disorder that frequently coexists with KS in advanced HIV [7]. Imaging demonstrated inguinal, iliac, and retroperitoneal lymphadenopathy. MCD commonly presents with generalized lymphadenopathy, Pancytopenia, and systemic inflammation [7]. This patient exhibited several of these features, including anemia, markedly elevated inflammatory markers, and extensive lymphadenopathy, alongside edema likely secondary to lymphatic obstruction. Notably, 63% of lymph nodes involved by MCD in HIV-positive patients showed coexisting KS in one study [9]. The National Institutes of Health/Infectious Diseases Society of America (NIH/IDSA) opportunistic infection (OI) guidelines recommend rituximab as first-line treatment for MCD (BII), with addition of liposomal doxorubicin for concurrent KS [3]. In people with HIV, multicentric lymphadenopathy with HHV-8 detected in blood or tissue supports clinical suspicion for MCD; however, confirmation requires excisional lymph node biopsy, which was not performed in this patient [3,7]. Early recognition is critical, as untreated MCD can lead to multi-organ failure and death [8].

## Conclusions

This case illustrates the life-threatening consequences of antiretroviral noncompliance, resulting in profound immunosuppression complicated by biopsy-proven oral KS with suspected concurrent MCD. The overlapping infectious, malignant, and inflammatory manifestations of advanced AIDS underscore the indispensable role of tissue-based diagnosis, as clinical suspicion alone is insufficient to distinguish between these entities. The shared pathogenic role of HHV-8 in KS and MCD, together with the possibility of secondary hemophagocytic lymphohistiocytosis in the presence of marked systemic inflammation, necessitates a high index of suspicion and coordinated multidisciplinary management. Sustained ART adherence remains the most critical intervention in preventing AIDS-defining complications and improving long-term outcomes.
